# Optimal Strategies for Mitigating Sudden Cardiac Death Risk in At-risk Patients with Structural Heart Disease

**DOI:** 10.19102/icrm.2018.090204

**Published:** 2018-02-15

**Authors:** Elaine Boey, Pipin Kojodjojo

**Affiliations:** ^1^Division of Cardiology, Ng Teng Fong General Hospital, National University Health System, Singapore

**Keywords:** Cardioverter-defibrillator, heart disease, sudden cardiac death

## Abstract

This article reviews the strategies used to mitigate sudden death risks in at-risk patients with structural heart disease. The roles of implantable and non-implantable technologies to prevent arrhythmic death are discussed.

## Introduction

Sudden cardiac death (SCD) is a leading cause of death in developed countries, affecting approximately 325,000 people in the United States annually, with an increasing incidence in the last decade.^1^ On average, fewer than 11% of SCD patients survive to hospital discharge.^1^ Having a history of heart disease is a major risk factor of SCD, with patients who have structural heart disease being seven times more likely to develop SCD than patients with structurally normal hearts.^1^ Implantable cardioverter-defibrillator (ICD) therapy can reduce mortality and is cost-effective in selected patient populations at risk for SCD. In this article, we review the application of strategies to prevent SCD in patients with structural heart disease and impaired ejection fraction (EF).

### Established indications and target populations

The first ICD implant was performed in 1980 and, since then, multiple large primary and secondary prevention trials have proven decisively that ICD reduces SCD and mortality.^2^ In turn, these trials form the foundation upon which international guidelines and appropriate use criteria are determined. Secondary prevention studies, conducted in the 1990s, examined the benefits of ICD in patients who had already aborted SCD or who had hemodynamically significant ventricular arrhythmias.^3–5^ Based on a meta-analysis of three key studies [ie, Antiarrhythmics Versus Implantable Defibrillators (AVID), Cardiac Arrest Study Hamburg (CASH), and the Canadian Implantable Defibrillator Study (CIDS)], ICD therapy was associated with a 28% relative risk reduction in total mortality, and a 50% risk reduction in arrhythmic death.^6^ In contrast, primary prevention studies have typically focused on at-risk patients with impaired left ventricular ejection fraction (LVEF) alone or in conjunction with other risk markers for sudden death.^7–10^ For instance, both the Multicenter Autonomic Defibrillator Implantation Trial I (MADIT-I) and Multicenter Unsustained Tachycardia Trial (MUSTT) studies enrolled patients with primary coronary artery disease, LVEF of 40% or less (35% or less in MADIT-I), spontaneous non-sustained ventricular tachycardia (VT), and inducible ventricular arrhythmias during electrophysiological studies (EPS).^8,9^ In MUSTT, patients were randomized to follow either an antiarrhythmic strategy, which included antiarrhythmic agents and, after at least one unsuccessful drug test, ICD implantation or no antiarrhythmic therapy. In MADIT-I, patients were assigned to receive either conventional medical therapy (of which more than three-quarters received antiarrhythmic agents) or an ICD. Both studies demonstrated that ICD therapy reduced the risk of overall mortality in excess of 54%, while antiarrhythmic pharmacological therapy did not improve survival. Subsequent studies were designed with fewer inclusion criteria: MADIT-II enrolled 1,232 patients with prior myocardial infarction and LVEF of 30% or less, whereas the Sudden Cardiac Death in Heart Failure Trial (SCD-HeFT) examined the role of ICD therapy in patients with both ischemic and non-ischemic cardiomyopathy (NICM), an LVEF of 35% or less, and a New York Heart Association (NYHA) class II/III heart failure status.^7,10^ Overall mortality was reduced by 31% and 23%, respectively, in MADIT-II and SCD-HEFT.

Of note, though ICDs have been proven to be more effective in reducing mortality **(Table 1)** than other common treatments used for cardiac disease, it should be mentioned that the life-saving potential of ICDs may have been underestimated by these landmark studies. Given that the risk associated with device implantation is front-loaded and that device longevity is typically more than seven years, the above-mentioned trials, which were frequently terminated prematurely, with median follow-up periods of less than four years, may have underestimated the lifesaving benefits of ICD and artificially inflated the numbers needed to treat to prevent one death.^11^

Still, on the basis of these landmark trials, international guidelines have recommended ICD therapy for the following patient groups who are on optimal medical therapy and who have a reasonable expectation of survival of more than one year.^12,13^ Some of the most common indications for ICD implantation for primary prevention purposes include (1) in cases of NICM or ischemic heart disease presenting at least 40 days post-myocardial infarction (MI) with an LVEF ≤ 35% and NYHA class II/III symptoms; (2) in patients with left ventricular (LV) dysfunction due to prior MI who are at least 40 days post-MI and who have LVEF ≤ 30% and NYHA functional class I; and (3) in those with non-sustained VT due to prior MI, LVEF ≤ 40%, and inducible sustained VT during electrophysiology study. Indications for ICD implantation for secondary prevention, on the other hand, include (1) patients who have survived ventricular fibrillation (VF) or hemodynamically unstable VT not due to a completely reversible cause; (2) patients with structural heart disease and spontaneous sustained VT, regardless of whether it is hemodynamically stable or unstable; (3) patients with VT with syncope who have an LVEF ≤ 40%; and (4) those with relevant, hemodynamically significant sustained VT or VF induced during electrophysiology study.

Interestingly, while traditional guidelines have recommended ICD therapy for use in NICM patients with symptomatic heart failure and depressed LVEF, a recent trial has rekindled the debate regarding how best to apply ICD therapy in NICM patients. The DANISH study randomized 556 NICM patients with symptomatic heart failure (NYHA class II/III), LVEF ≤ 35%, and elevated brain natriuretic peptide levels to receive either ICD therapy or routine medical care.^14^ Of note, this contemporary cohort were treated optimally with proven heart failure therapies, with more than 92% receiving β-blockade and renin-angiotensin system inhibitors, in excess of 57% on mineralocorticoid antagonists, and 93% of patients with left bundle branch block in excess of 150 ms receiving cardiac resynchronization therapy (CRT). Remarkably, in both arms, 58% of patients were receiving CRT, a rate much higher than that typically expected in a heart failure cohort. While arrhythmic deaths (sudden cardiac death or VT/VF) were reduced via the use of ICD therapy, cardiovascular deaths (which include death from heart failure, stroke, and other cardiovascular conditions) and overall mortality (from any cause) remained unchanged over a follow-up period of more than 67 months. Prior to this study, no single ICD trial enrolling NICM patients has shown a statistically significant reduction in mortality, although there was a trend towards significance in the DEFINITE and SCD-HEFT trials.^7,15^ In both studies, the percentages of patients on optimal pharmacological therapies were lower, and no patients received CRT, including those patients with a wide QRS for whom CRT would be indicated. Despite this, a recent meta-analysis of NICM patients randomized in primary prevention ICD trials, including DANISH, found that ICD placement remains associated with a 21% reduction in overall mortality and a 53% reduction in arrhythmic death.^16^ Taken in conjunction with one another, these findings confirm NICM patients remain at risk from SCD, although their burden of arrhythmic death is comparatively lower than that of subjects with ischemic heart disease. It also raises doubts whether the simplistic markers of SCD such as low EF, validated for ischemic heart disease, should be applied to select NICM patients at risk of SCD. There is a growing body of literature that sophisticated measures such as presence and distribution of enhancement, representative of myocardial scarring, on gadolinium-enhanced cardiac magnetic resonance imaging would be a better risk predictor for NICM.^17,18^

#### At-risk populations for whom ICD therapy has not been beneficial

It may seem intuitive given the life-saving benefits of ICDs in patients with impaired LVEF would be conferred to patients early post-MI. DINAMIT randomized 674 patients between six and 41 days post-MI, LVEF ≤ 35%, and abnormal autonomic parameters namely depressed heart rate variability or elevated average 24-hour heart rates on Holter monitoring to either ICD or conventional therapy.^19^ Over a mean follow-up of 30 months, there was no difference with overall mortality between the groups. While arrhythmic deaths were reduced by 58%, there was a 75% increase in non-arrhythmic deaths in the ICD group, resulting in no net benefit. The same conclusions were reached by the IRIS study, which randomized 898 patients who were five to 31 days post-MI, and who had LVEF ≤ 40%, elevated heart rate on electrocardiogram (ECG), and/or non-sustained VT on Holter monitoring, to receive an ICD implant or medical therapy alone.^20^ Similarly, ICD therapy was associated with a 45% reduction in arrhythmic deaths, but a 92% relative risk in non-arrhythmic deaths, resulting in no net benefit. An important feature of IRIS was that 77% of patients received reperfusion therapies, with three-quarters receiving percutaneous coronary intervention, consistent with contemporary clinical practice. In the early period after MI, non-arrhythmic causes of SCD such as recurrent MIs and myocardial rupture may account for 48% of mortality, and the proportion of sudden death attributable to arrhythmic versus non-arrhythmic causes only increases after months.^21^ Post hoc analysis of MADITII data also supports this hypothesis, with the survival benefit of ICD showing time-dependency and being more effective in patients with remote (> 18 months) versus recent MI (< 18 months).^22^ In conjunction, the implantation of ICD early after a MI does not necessarily save lives.

It is also well recognized that there are certain high-risk patient subgroups in whom the role of device therapy remains poorly defined. This includes patients with NYHA class IV heart failure symptoms, those with end-stage renal failure on renal replacement therapies, and adults with corrected congenital heart disease. In the interim, the management of these patients should be individualized on a case-by-case basis.

#### Cardiac resynchronization therapy

A full review of CRT is beyond the scope of this manuscript, but in eligible patients with broad QRS duration (QRSd) of more than 120 ms, CRT alone has been shown to confer a mortality benefit, in addition to improving LVEF and quality of life and reducing the need for heart failure hospitalizations.^23,24^ The extent of improvement is dependent on patient characteristics, with female patients and those individuals with left bundle branch block, wider QRS complexes, and/or more severe heart failure symptoms deriving more benefit.^23–26^ The CARE-HF study randomized NYHA class III or IV heart failure patients with an LVEF of 35% or less and a QRSd of 120 ms or greater to either optimal medical therapy or a CRT pacemaker. CRT use resulted in a 36% reduction in all-cause mortality and a 52% decrease in unplanned heart failure hospitalizations.^24^ Similar results were found in the COMPANION study, whereby NYHA class III or IV heart failure patients with LVEF of 35% or less, QRSd of 120 ms or more and a PR interval of more than 150 ms were randomized to receive optimal medical therapy, CRT alone, or CRT in combination with an ICD.^23^ The provision of CRT alone was associated with a marginally significant (24%) reduction in all-cause mortality (p = 0.06). The addition of ICD therapy improved relative survival by a further 12%. The degree of reduction in hospitalizations for heart failure and cardiovascular causes was statistically similar in both CRT groups.

#### Subcutaneous implantable cardioverter-defibrillators

With an increasing aging population, improved survival among patients with complex heart disease, and easier access to ICDs, a greater number of patients are living with ICDs. While the use of ICDs has shown a clear survival benefit in the above-mentioned patient populations, the use of transvenous leads has long been recognized to be linked with periprocedural and long-term complications, such as venous thrombosis, lead dislodgement, cardiac perforation, and lead failures. If necessary, lead extraction, especially of mature leads, exposes patients to further procedural risks, including vascular damage requiring surgery and even death. Furthermore, the anatomy in certain patients may be unsuitable for transvenous lead placement due to the absence of upper limb venous access, mechanical tricuspid valves, and congenital heart disease with a univentricular physiology.

**Figure 1:**
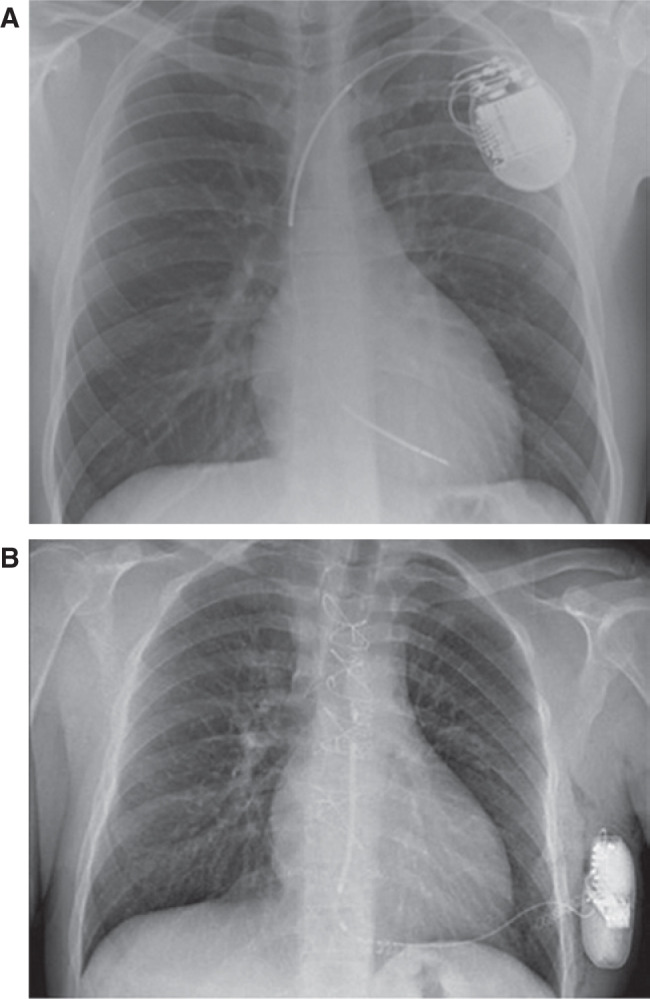
Chest radiographs demonstrating the differences between transvenous and subcutaneous ICD systems. **A:** Transvenous ICD. **B:** S-ICD.

To eliminate the need for venous access and potentially avoid lead-related complications, the first subcutaneous ICD (S-ICD) was developed and implanted in 2008, which consisted of an extra-cardiac, extra-thoracic, fully subcutaneous system with a pulse generator implanted in the left axillary position and a single lead containing a defibrillation coil housed between two sensing electrodes implanted parallel and lateral to the sternum **(Figure 1)**.^27^ The pulse generator functions both as the third sensing electrode and as a part of the defibrillation pathway, allowing for the entire system to provide three sensing vectors and two shock polarities.

The main advantage of S-ICD use is the redundancy of vascular access and lead-related complications. S-ICD lead placement does not require stylets and, hence, leads do not have a lumen, giving it greater tensile strength. Coupled with the fact that S-ICD leads are also subjected to less mechanical stress than their transvenous counterparts as imposed by the beating heart, the longevity of the S-ICD lead is expected to be greater than transvenous leads. Furthermore, S-ICD implantation is performed based on anatomical landmarks, eliminating the need of fluoroscopy and exposure to radiation. Subsequent lead extraction is relatively easier than that of transvenous lead systems, although the oldest S-ICD systems are still less than eight years old, a fact that limits our clinical experience with the removal of mature subcutaneous leads. By the nature of its method of implantation, S-ICD implantation also avoids periprocedural complications of pericardial effusion and pneumothorax. The rate of complications of hematoma and device and lead displacement have been found to be half that of conventional ICDs.^28^

An early study by Bardy et al. found that the above device configuration is as effective as conventional ICDs in terminating VT, although a significantly higher energy requirement (36 J versus 11 J) was needed.^27^ Despite the obvious strengths, however, the usage of S-ICDs presents a different set of complications. In comparison with a conventional ICD, S-ICDs have a larger pulse generator, which increases the risk of tissue necrosis.^29^ Additionally, with repeated discharges, they thus will exhibit a shorter estimated battery longevity due to the higher energy deliveries, and their costs remain significantly more than those of a single-chamber ICD.^30^

Owing to the position of the sensing electrodes, the first generation of S-ICDs was noted to be more susceptible to inappropriate shocks resulting from T-wave oversensing, supraventricular arrhythmias, myopotentials, and double counting from bundle branch block.^31^ While simulation studies have found S-ICD use to be more specific in terms of supraventricular arrhythmia discrimination than transvenous ICDs, in practice, the inappropriate therapy rate of S-ICD use is much higher than that of conventional ICDs, mostly due to T-wave oversensing.^27,32–34^ Subsequent modifications such as the use of a suture sleeve at the xiphoid incision to prevent lead displacement, software updates to improve detection algorithms, and the usage of dual-zone algorithms have reduced the number of inappropriate shocks significantly.^28,34,35^ In dual-zone programming, all detections within the programmed shock zone, regardless of their characteristics, are considered shockable. Within the slower conditional shock zone, the QRS width and morphology of the detected signal is compared with a reference ECG stored during sinus rhythm. In addition, beat-to-beat morphology variations are also examined and, based on these three parameters, the S-ICD determines whether therapy should be delivered.^36^ Confirmation of a shockable rhythm is ensured separately prior to capacitor charging and shock delivery.

Preimplant ECG screening with patients in different postures is crucial to identifying patients unsuited for S-ICD use, further lowering the rate of inappropriate therapy. Approximately 7% to 10% of patients fail the screening criteria, with a greater proportion being those with hypertrophic cardiomyopathy or congenital heart disease.^37–39^ It is controversial as to whether the exercise testing used to screen patients believed to be at high risk for T-wave oversensing, such as those with right bundle branch block, abnormal repolarization on the ECG, or those who are physically very active in daily life, provides any additional information for patient selection.^40,41^ While the S-ICD is able to provide antibradycardia pacing for up to 30 seconds after a shock is delivered, it is unsuitable for use in patients who require chronic pacing therapy or antitachycardia pacing. Should an S-ICD recipient subsequently develop an indication for pacing, an additional device is necessary. While the coexistence of a leadless pacemaker and an S-ICD has been reported in isolated case reports, longer-tem data and the ability to provide anything beyond right ventricular pacing are lacking.^42^ In view of the above limitations, an S-ICD may be a preferred option for younger patients who are expected to have an increased lifetime risk of complications with transvenous ICD use, as well as those who are at an increased risk of infection, those who have had previous lead complications or vascular access issue, and/or those who do not have a requirement for bradycardia or antitachycardia pacing. Ongoing clinical trials such as MADIT S-ICD (NCT02787785) and S-ICD Brugada (NCT02344277) are expected to better identify patient subgroups who might benefit most from the use of S-ICD technologies. To date, however, no randomized controlled trials have been completed regarding S-ICD use and, unlike in the case of transvenous ICDs, no clear mortality benefit has been demonstrated. However, given our understanding of the life-saving benefits of ICD therapy, a randomized controlled study of S-ICD use versus traditional medical therapy in patients who are already eligible for ICD implantation would be ethically impossible. A comparative study between S-ICD and transvenous ICD use, ATLAS S-ICD (NCT02881255), is underway, and is designed primarily to examine lead-related perioperative complications, although prespecified secondary endpoints include mortality, successful defibrillation, and inappropriate shocks. Such data would strongly validate the ongoing “real-world” practice of implanting S-ICDs instead of transvenous ICDs in patients eligible for both technologies.

#### Wearable cardiac defibrillators

Although ICDs have proven efficacy, there exist certain populations of at-risk individuals who are temporarily unsuitable to undergo ICD implantation, or who may not fulfill the criteria for ICD implantation in time due to recovery of myocardial dysfunction. These patients include (1) those who have undergone lead and device extraction due to infection, and who are awaiting completion of antibiotic therapy prior to reimplantation; (2) those with a new diagnosis of NICM who are undergoing a trial of guidelines-directed medical therapy, to allow for recovery; and (3) those with coronary artery disease acutely post-revascularization, who have high-risk features such as severe LV dysfunction or non-sustained ventricular arrhythmias. During this interim period, they remain at risk of SCD and, therefore, a temporalizing solution such as an external wearable cardiac defibrillator (WCD) would be desirable.

The WCD is a vest equipped with sensing and defibrillation electrodes that is typically worn under the patient’s clothing. It monitors their cardiac rhythm continuously, transmits information on the patient’s arrhythmias as needed, and delivers a shock if required. It is programmable to have several zones with variable response times and shock energy. The WCD has been previously shown to be more prone to administering inappropriate therapy due to artifacts. Thus, countermeasures such as noise-reducing algorithms, sensing functions to identify poor electrode contact, and an alarm system that allows for patients to participate in the prevention of inappropriate therapy have been developed in order to reduce the incidence of inappropriate shocks. Unlike an ICD, the WCD is unable to provide backup pacing or antitachycardia pacing functions.

Trials using the WCD in early post-MI patients have reported appropriate shocks, mostly in the first month after MI, with more than 80% shock success, in both revascularized and unrevascularized patients, suggesting the WCDs can detect and treat arrhythmias successfully, potentially reducing arrhythmic morality during this period.^43^

However, it is important to keep in mind that the true benefits of WCD use have yet to be proven. Though research has shown that WCDs deliver therapy in patients to abort VT/VF with a high first-shock success rate, the incidence of life-threatening arrhythmias necessitating defibrillation in these studies has been low. In a cohort of post-MI patients with EF ≤ 35%, the incidence of subjects experiencing WCD shocks for VT/VF was 3% and, among patients with NICM, only 1% received appropriate shocks.^44,45^ In a single-center series of 254 NICM patients prescribed WCD use over a period of 10 years, no patients received an appropriate shock.^46^ Another study involving a mixed patient population who qualified for WCD use showed that only 2.7% of arrhythmias lasted more than 25 seconds, and that the majority of them were self-terminating. The rate of appropriate shocks was 1.58 per 100 patient-months, and the rate of inappropriate shocks was 0.99 per 100 patient-months.^47^ Furthermore, as seen in the DINAMIT and IRIS studies, even having a defibrillator implanted in situ in the early post-MI period did not seem to confer an overall mortality benefit. As such, while WCDs have shown themselves to be clinically functional, their true benefits in terms of longer-term outcomes and cost reduction remain undetermined. Of note, patient non-compliance with wearing the vest can potentially lead to 10% to 30% of study participants discontinuing device use due to comfort or lifestyle reasons, further impacting clinical efficiency and cost-effectiveness.^47,48^

Despite these uncertainties, however, it is undeniable that WCDs have a niche that straddles the gap between having an ICD implanted and going sans cardiac protection. The precise role of WCDs is expected to become clearer with the making of technological modifications to improve patient comfort/compliance and the conduction of studies aimed at providing data on long-term outcomes and ideal patient subgroups who might best benefit from the use of these devices.

## Community preparedness

Most cases of SCD occur in the general population, and largely in patients whose LVEF scores do not fulfill the current criteria for (> 40%) for ICD therapy. In fact, many of these individuals are asymptomatic, and do not have or have never had a formal diagnosis of cardiac disease.^49^ Within the general population, parameters to identify potential SCD victims lack sensitivity, while the implantation of ICDs in this large group would be impractical and unlikely to be cost-effective.

In this setting, community preparedness and the capability to provide prompt bystander or first responder cardiopulmonary resuscitation (CPR) is critical. The links in this “chain of survival” represent the performance of early assessment and the use of early basic life support, early defibrillation, and early advanced life support, respectively.^50^ Bystander-performed CPR has been seen to improve survival following SCD by two to four times,^51–53^ and earlier defibrillation administration performed within three to five minutes of SCD onset improves survival by 50% to 70%.^54–56^ Each minute of delay in defibrillation decreases the chance of survival by 10%.^57,59^ However, despite the clear association between early response and survival, it has been previously found that only 15% to 30% of SCD victims in the community receive bystander CPR, while only 3% received defibrillation using an automated external defibrillator (AED).^59,60^ Ideally, in urban settings, bystander CPR should always be initiated earlier than any emergency response team’s arrival on the scene. There has been a multitude of approaches employed to improve bystander CPR rates.^61^

With smartphones becoming one of the most common devices in the community, smartphone applications have the potential to impact public education, including with respect to occurrence of SCD onset and AED locations. A study in Sweden found that bystander CPR incidence increased by 14% when a mobile phone system was employed to alert bystanders to a nearby collapse.^55^ A smartphone with an accelerometer can even provide real-time feedback on the quality of CPR efforts, potentially improving outcomes. Increasing the number of bystanders who are capable of performing CPR and competent in the use of AEDs by mass public education campaigns would favorably impact the timing and delivery of early care. Public awareness regarding CPR has already increased through mass training events, mandatory CPR testing, CPR training in schools, and the introduction of mobile phone applications. The provision of ambulance dispatcher assistance to bystanders performing CPR has also been found to approximately double bystander CPR performance rates, with a consequent improvement in 30-day survival rates.^62^

Increasing public awareness regarding CPR performance also means that more people will likely be able to operate and use an AED effectively. Expanding the accessibility of AEDs has further improved the chain of survival. The PAD trial showed that having more AEDs in the community improved survival twofold.^63^ More recently, the concept of having a drone network designed to deliver AEDs to the site of an out-of-hospital cardiac arrest has been modeled, with results demonstrating that such a strategy could result in the quicker availability of AEDs.^64^ Further field testing is required to validate this interesting concept. One caveat, however, is that although having shorter AED response times should logically result in reduced SCD mortality, another study found that the deployment of AEDs in the homes of patients early after anterior MI did not result in reduced mortality compared with training family members in basic life support methods, suggesting that multiple factors influence the use of AEDs in the community.^65^

Undoubtedly, prevention is better than a cure. Primary and secondary preventative measures of coronary heart disease, to which 80% of SCD episodes are attributed to, particularly in emerging countries, are vital to implement. A greater emphasis on delivering guidelines-directed care—such as by ensuring the appropriate use of statins, β-blockers, and angiotensin-converting-enzyme inhibitors; tackling obesity; achieving optimal diabetic control; prompting the cessation of smoking habits in those patients who are vulnerable; and pushing for the adaptation of widespread positive lifestyle measures to reduce the overall risk of SCD—should be considered.

## Figures and Tables

**Table 1:** The Number of Patients that Must be Treated by Common Cardiovascular Interventions to Prevent One Death.

Intervention (Landmark Study)	Sample Size	Number of Patients that Must be Treated to Prevent One Death
Secondary prevention ICD (AVID)^3^	1,232	9
Primary prevention ICD (SCD-HeFT)^7^	2,521	15
Primary prevention ICD (MADIT-II)^10^*	1,016	18
CRT (COMPANION)^23^†	1,520	17
Aspirin (ISIS-2)^66^	17,000	38
Enalapril (SOLVD)^67^	2,600	22
Simvastatin (4S)^68^	4,444	29
Primary PCI (meta-analysis)^69^	7,739	43
Cardiac rehabiliation (meta-analysis)^70^	7,683	72
Primary prevention ICD (MADIT-II) – extended follow-up^71^*	1,016	8

*The number needed to treat out of 18 to prevent one death in the MADIT-II study was based on 1.5 years of median randomized follow-up, during which patients were assigned to receive either conventional medical therapy or an ICD implant. Given that ICD battery life is typically more than four years, the premature termination of this study would lead to the underestimation of the lifesaving potential of ICDs. When the follow-up period of MADIT-II was extended for all participants, including allowing for a crossover of medically treated patients to receive ICD, the number of patients needed to treat to prevent one death decreased from 18 to eight.

†The data presented are based on results from the CRT-P arm of the COMPANION trial, with a median follow-up of only 16.2 months, and therefore are also underestimated.

ICD: implantable cardioverter-defibrillator; AVID: Antiarrhythmics Versus Implantable Defibrillators; SCD-HeFT: Sudden Cardiac Death in Heart Failure Trial; MADIT: Multicenter Automatic Defibrillator Implantation Trial; CRT: cardiac resynchronization therapy; COMPANION: Comparison of Medical Therapy, Pacing, and Defibrillation in Chronic Heart Failure; ISIS-2: Second International Study of Infarct Survival; SOLVD: Studies of Left Ventricular Dysfunction; 4S: Scandinavian Simvastatin Survival Study; PCI: percutaneous coronary intervention.
